# Cattle phenotypes can disguise their maternal ancestry

**DOI:** 10.1186/s12863-017-0523-5

**Published:** 2017-06-26

**Authors:** Kanokwan Srirattana, Kieren McCosker, Tim Schatz, Justin C. St. John

**Affiliations:** 1 Centre for Genetic Diseases, Hudson Institute of Medical Research, Clayton, VIC 3168 Australia; 2 Department of Molecular and Translational Sciences, Monash University, Clayton, VIC 3168 Australia; 3 Department of Primary Industry and Resources, Darwin, NT 0800 Australia

**Keywords:** Mitochondrial DNA, Phylogenetics, Cattle, Embryo development, Animal breeding

## Abstract

**Background:**

Cattle are bred for, amongst other factors, specific traits, including parasite resistance and adaptation to climate. However, the influence and inheritance of mitochondrial DNA (mtDNA) are not usually considered in breeding programmes. In this study, we analysed the mtDNA profiles of cattle from Victoria (VIC), southern Australia, which is a temperate climate, and the Northern Territory (NT), the northern part of Australia, which has a tropical climate, to determine if the mtDNA profiles of these cattle are indicative of breed and phenotype, and whether these profiles are appropriate for their environments.

**Results:**

A phylogenetic tree of the full mtDNA sequences of different breeds of cattle, which were obtained from the NCBI database, showed that the mtDNA profiles of cattle do not always reflect their phenotype as some cattle with *Bos taurus* phenotypes had *Bos indicus* mtDNA, whilst some cattle with *Bos indicus* phenotypes had *Bos taurus* mtDNA. Using D-loop sequencing, we were able to contrast the phenotypes and mtDNA profiles from different species of cattle from the 2 distinct cattle breeding regions of Australia. We found that 67 of the 121 cattle with *Bos indicus* phenotypes from NT (55.4%) had *Bos taurus* mtDNA. In VIC, 92 of the 225 cattle with *Bos taurus* phenotypes (40.9%) possessed *Bos indicus* mtDNA. When focusing on oocytes from cattle with the *Bos taurus* phenotype in VIC, their respective oocytes with *Bos indicus* mtDNA had significantly lower levels of mtDNA copy number compared with oocytes possessing *Bos taurus* mtDNA (*P* < 0.01). However, embryos derived from oocytes with *Bos indicus* mtDNA had the same ability to develop to the blastocyst stage and the levels of mtDNA copy number in their blastocysts were similar to blastocysts derived from oocytes harbouring *Bos taurus* mtDNA. Nevertheless, oocytes originating from the *Bos indicus* phenotype exhibited lower developmental potential due to low mtDNA copy number when compared with oocytes from cattle with a *Bos taurus* phenotype.

**Conclusions:**

The phenotype of cattle is not always related to their mtDNA profiles. MtDNA profiles should be considered for breeding programmes as they also influence phenotypic traits and reproductive capacity in terms of oocyte quality.

**Electronic supplementary material:**

The online version of this article (doi:10.1186/s12863-017-0523-5) contains supplementary material, which is available to authorized users.

## Background

Mitochondria play important roles in energy production via the electron transport chain, cell death signalling, and the control of stress responses (reviewed by [1]). Mitochondria have their own genome, mitochondrial DNA (mtDNA) which is maternally inherited [2]. The bovine mitochondrial genome is 16.3 kb in size and encodes 13 of the subunits of the electron transfer chain, 22 tRNAs and 2 rRNAs [3]. Its replication and copy number are cell type specific [4] with oocytes having the largest population of mtDNA of any cell type [5, 6]. MtDNA replication is controlled by nuclear-encoded transcription and replication factors that translocate to the mitochondrion and interact with the D-loop region to drive first transcription and then replication of mtDNA [7]. The D-loop is not only the control region [8], but it also possesses two hypervariable regions that are used by molecular geneticists to map the maternal ancestral inheritance of the mitochondrial genome [9].

Over billions of years, the mitochondrial genome has evolved as individuals have adapted to the environments that they have chosen to inhabit [10, 11]. Mitochondrial haplotypes are clusters of mitochondrial genomes that collectively define the maternal lineages of groups of individuals. Mitochondrial haplotypes have also been shown to predispose individuals to and protect them against a number of diseases and phenotypic traits. For example, they are associated with tolerance to heat [12], longevity [13], fertility and litter size [14], time to pregnancy, and milk and meat quality [15].

As climate change affects the ability of livestock to function efficiently in their environments [16], it has been proposed that a number of assisted reproductive technologies, such as somatic cell nuclear transfer, could be used to generate animals to suit the affected environments. This would involve the generation of founder animals that contain appropriate nuclear genotypes matched with desirable mitochondrial haplotypes that would then be introduced into breeding programs [17, 18]. Indeed, as an example, the northern regions of Australia, which have a tropical climate, are more suitable for populations of cattle that exhibit heat tolerance, such as some *Bos indicus* cattle that originated from Africa and Asia. However, the British and European breeds, such as *Bos taurus* cattle, would tend to prosper more in the more temperate, southern regions of Australia, where the climate is cooler in winter. However, if mtDNA haplotypes are indicators of, for example, tolerance to heat or adaptability to a specific environment, then assessment of these animals would provide an indication of whether, indeed, the appropriate genetics is matched to the environment that the offspring are located in. In support of this, a previous report in human cells showed that different mtDNA haplotypes had different levels of expression for the nuclear-encoded heat shock protein genes, *HSP60* and *HSP75*, which are two key components of the mitochondrial heat stress response machinery [19]. Moreover, *HSP75*, also known as *TRAP1*, has been reported to be a candidate gene for body temperature regulation in cattle [20].

Furthermore, it has been demonstrated that there is a relationship between tolerance to heat and oocyte developmental competence, i.e. the ability of an oocyte to mature, fertilise and develop into an embryo [21] and calving interval in cattle [22]. Likewise, in commercial pigs, there is a relationship between mtDNA haplotypes, litter size and developmental competence with oocyte mtDNA copy number being a key determiner of oocyte developmental competence [14].

As mtDNA haplotypes have not been rigorously considered as a selection tool by the livestock breeding industries when migration and breeding programs are planned, the performance of cattle could potentially be improved by ensuring that they have the appropriate mtDNA for their environment. Indeed, it is anticipated that *Bos taurus* cattle would be suited to southern Australia in terms of both their chromosomal and mtDNA content and that *Bos indicus* cattle would be more suited to northern Australia if their genotypes and mtDNA haplotypes were appropriately matched to suit the environment. In this study, we have investigated the mtDNA genetic profiles of cattle that are located in Victoria (VIC), southern Australia and cattle from the Northern Territory (NT) to determine whether the cattle have the genetics associated with the chromosomal and mitochondrial genomes from their relevant phenotypes. Our outcomes, generated using sequencing technology, demonstrate that, whilst a number of cattle in southern Australia of the *Bos taurus* phenotype have *Bos taurus* chromosomes and mitochondrial genomes, some cattle also have *Bos indicus* mtDNA haplotypes. Likewise, in a subgroup of cattle located in the Northern Territory, a mixture of cattle of the *Bos indicus* phenotype with either *Bos indicus* or *Bos taurus* mtDNA haplotypes were identified. Furthermore, when we investigated the relationship between oocyte developmental rates and oocyte mtDNA copy number, we determined that the developmental rates of oocytes derived from the *Bos indicus* phenotype with *Bos indicus* mtDNA were lower than that of the *Bos taurus* phenotype, due to low oocyte mtDNA copy number. This suggests that the movement of cattle around Australia is not necessarily supportive of the genetic fitness of these animals to adapt to the regions in which they are located and, indeed, that they may have the wrong mitochondrial genetics to support development and productivity.

## Methods

All chemicals and reagents were purchased from Sigma-Aldrich (St. Louis, MO, USA) unless otherwise specified.

### Samples

Samples were collected from 346 individuals (225 cattle with a *Bos taurus* phenotype and 121 cattle with a *Bos indicus* phenotype) for mtDNA genotyping by mtDNA sequencing. The samples consisted of: 1) oocytes (94 samples) and ovarian tissue (131 samples) collected from slaughtered cattle with a *Bos taurus* phenotype (Angus phenotype) from 2 local abattoirs in the southern part of Australia, VIC; and 2) hair samples (UQ, SJ, Ba, Sta) from cattle with a *Bos indicus* phenotype (121 samples) from research and commercial beef breeding cattle herds at 4 different beef breeding stations in the NT of Australia, as detailed in (see Additional file 1: Table S1).

For mtDNA copy number and embryo development analysis, ovaries were collected from slaughtered cattle with a *Bos taurus* phenotype and oocytes isolated and activated to generate embryos.

### Relationship between breeds of cattle

To determine how representative mtDNA haplotypes were of different breeds of cattle, CLC Genomics Workbench version 9.5.1 (CLC Bio, Aarhus, Denmark) was used to align and construct a phylogenetic tree from 56 full mitochondrial genome sequences obtained from the NCBI database (data obtained on 31^st^ July 2016), (see Additional file 2: Table S2). The phylogenetic tree was constructed using the maximum likelihood method [23]. To determine the best nucleotide substitution model for maximum likelihood tree construction, model testing was performed [24], as we have previously described [14]. We tested the Jukes-Cantor, Felsenstein 81, Kimura 80, Hasegawa-Kishino-Yano (HKY) and general time reversible (GTR) models, using four different statistical analyses: the Hierarchical likelihood ratio test; Bayesian information criterion; Minimum theoretical information criterion; and Minimum corrected theoretical information criterion. The GTR model [25] was deemed to be the best by most statistical tests. The reliability of the branching order was estimated from 1000 bootstrap replicates. For analysis efficiency, an initial tree was constructed first using the neighbour-joining method from aligned mtDNA sequences. The maximum likelihood tree was then determined from the initial tree to obtain optimum topology, otherwise known as the NJML approach, which is a hybrid algorithm to improve analysis efficiency [26].

### MtDNA genotyping of cattle

To determine mtDNA profiles of cattle in VIC and NT, total DNA was extracted from hair and ovarian samples (see section ‘Samples’) using the ISOLATE II Genomic DNA Kit (Bioline, Alexandria, NSW, Australia), and from oocytes at the metaphase II (MII) stage (see section ‘Samples’) using the QIAamp DNA Micro Kit (Qiagen, Hilden, Germany), according to the manufacturer’s instructions. As the D-loop region of the mitochondrial genome has been used to study diversity, phylogenetic relationships and origin among species [27–31], the D-loop region was amplified by polymerase chain reaction (PCR). The 50 μl reaction mixtures consisted of 50 ng DNA sample or 10 μl oocyte DNA solution, 1× buffer (Bioline), 1.5 mM MgCl_2_ (Bioline), 1 mM dNTP mix (Bioline), 2.5 U DNA polymerase (BIOTAQ™, Bioline) and 0.5 μM of each primer (F: CACCATCAACCCCCAAAGCT, R: CCTGAAGAAAGAACCAGATG [32]). The amplification conditions were as follows: 95 °C for 5 min followed by 35 cycles at 94 °C for 30 s, 55 °C for 30 s and 72 °C for 45 s. The final step was 72 °C for 5 min. The PCR products were purified using the ISOLATE II PCR and Gel Kit (Bioline), according to the manufacturer’s instructions, and were sequenced by Capillary Electrophoresis using the ABI PRISM® BigDye Terminator Cycle Sequencing Ready Reaction Kit (Applied Biosystem, Foster City, CA, USA) on an ABI 3130xl Genetic analyzer (Applied Biosystem, Hitachi, Tokyo, Japan) by the Monash Health Translation Precinct Biomedical Genomics Facility, as described in [14].

The D-loop sequences were aligned and clustered with the cattle D-loop mtDNA reference sequences obtained from the NCBI database (AF492350 Zwergzebu, AY126697 Nellore, AY676857 Angus, DQ124418 Holstein, JN817350 Chianina, and KX575711 Sahiwal; data obtained on 31^st^ July 2016), using the CLC Genomics Workbench, as previously described (see section ‘Relationship between breeds of cattle’). A maximum likelihood phylogenetic tree was constructed from an initial tree constructed by the neighbour-joining method and the GTR as nucleotide substitution model [25] with 1000 bootstrap replicates.

### Oocyte preparation and embryo production

Briefly, cumulus oocyte complexes (COCs) were aspirated from 2 to 3 mm follicles from abattoir derived ovaries. The COCs were then cultured in in vitro maturation (IVM) media (TCM199 supplemented with 10% Fetal Bovine Serum (Gibco, Grand Island, NY, USA), 50 IU/ml hCG (Chorulon®, Intervet, Bendigo East, VIC, Australia), 0.5 μg/ml FSH (Folltropin®-V, Bioniche Animal Health, Belleville, ON, Canada), 1 μg/ml 17ß-estradiol) at 38.5 °C under humidified atmosphere of 5% CO_2_ in air for 19 h. Cumulus cells were then removed from the COCs by repeated pipetting in 0.2% hyaluronidase. MII oocytes exhibiting the first polar body were activated using 7% ethanol for 5 min at room temperature followed by 1.25 μg/ml cytochalasin D and 10 μg/ml cycloheximide at 38.5 °C under humidified atmosphere of 5% CO_2_, 5% O_2_ and 90% N_2_ for 5 h. After activation, the embryos were cultured in CR1aa medium (20 embryos per 100 μl CR1aa medium) [33] at 38.5 °C under humidified atmosphere of 5% CO_2_, 5% O_2_ and 90% N_2_ for 7 days. 50 μl of CR1aa medium was replaced at days 3 and 5 of culture.

### MtDNA copy number analysis in oocytes and embryos

Primers for assessing mtDNA copy number were designed from the consensus sequences for *Bos taurus* and *Bos indicus* mtDNA (DQ124418 Holstein, AY676857 Angus, AY126697 Nellore and AF492350 Zwergzebu; data obtained on 31^st^ July 2016) covering the NADH dehydrogenase subunit 2 (*ND2*) region to ensure that the primers were specific to all mtDNA profiles to prevent amplification bias. Total DNA was extracted from oocytes at the MII stage and embryos at the blastocyst stage using the QIAamp DNA Micro Kit, as described above. The mtDNA target sequence was quantified by real-time PCR using a Rotorgene 3000 real-time machine (Corbett Life Science, Sydney, NSW, Australia). The quantification of mtDNA was performed in 20 μl volumes of PCR mixtures containing 1× SensiMix™ SYBR Hi-ROX (Bioline), 0.25 μM of each primer (F: TATACGACTCACGTATTCTACC, R: CTTTGAAGGCTCTTGGTCTG) and 2 μl of oocyte or embryo DNA solution. PCR amplification was carried out at 95 °C for 15 min, followed by 45 cycles of 95 °C for 15 s, 55 °C for 15 s and 72 °C for 15 s. The final elongation step was performed at 72 °C for 15 s. The fluorescence signal was obtained via the FAM/Sybr channel at the elongation phase. From the first fluorescence signal, melting curve data were generated by heating PCR products from 47 to 98 °C, holding for 4 s at each step. These data were used to determine the second extension phase, which was set at the temperature just before the start of the melt curve phase to exclude the effects of primer-dimerisation. The second extension phase was set at 74 °C for *ND2* for 15 s and the second fluorescence signals were acquired.

To generate standards for real-time PCR, the *ND2* region was amplified from total DNA obtained from Holstein fibroblast cells. The PCR products were purified using the Isolate II PCR Gel Kit (Bioline), according to the manufacturer’s instructions, and were used as standards. For each real time PCR reaction, a standard curve was generated from 10-fold serial dilutions (10^−1^ to 10^−8^). Each sample was analysed in triplicate to obtain the mean number of mtDNA copies.

## Results

### Phylogenetic relationship between breeds of cattle

**Fig. 1:**
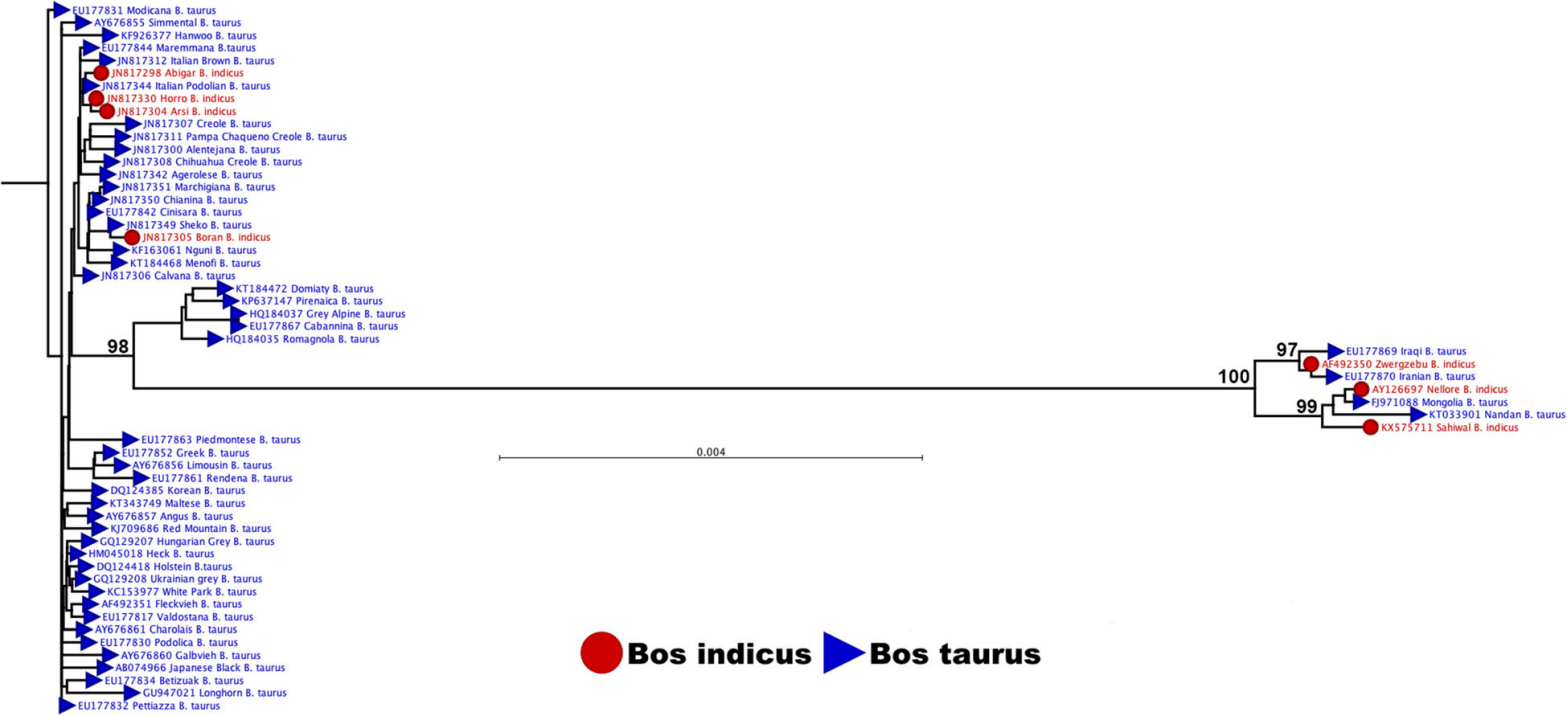
Phylogenetic tree constructed from the complete mitochondrial genomes of different breeds of cattle obtained from the NCBI database. The maximum likelihood phylogenetic tree was constructed by the general time reversible model with 1000 bootstrap replicates using the CLC Genomics Workbench version 9.5.1. 56 full mitochondrial genome sequences from different breeds of cattle obtained from the NCBI database (data obtained on 31^st^ July 2016) were analysed. Cattle with *Bos indicus* and *Bos taurus* phenotypes are represented in *red* and *blue*, respectively. Numbers above branches are bootstrap values >95%

In this study, 56 full mitochondrial genome sequences from different breeds of cattle were obtained from the NCBI database (see section ‘Relationship between breeds of cattle’). There were 49 sequences from *Bos taurus* breeds and 7 sequences from *Bos indicus* breeds. The phylogenetic tree showed two distinct mtDNA lineages which could be interpreted as evidence of two separate maternal origins, *Bos taurus* mtDNA and *Bos indicus* (Fig. 1). However, 4 (Iraqi, Iranian, Mongolia and Nandan) out of the 49 sequences from cattle exhibiting a *Bos taurus* phenotype clustered with the *Bos indicus* mtDNA sequences. Moreover, 4 (Abigar, Horro, Arsi and Boran) out of the 7 sequences from cattle with a *Bos indicus* phenotype clustered with the *Bos taurus* mtDNA sequences. These results indicate that cattle mtDNA profiles are not always indicative of their phenotype.

### Phylogenetic relationship between cattle from the northern Territory and Victoria

In order to determine the extent to which cattle mtDNA profiles are indicative of their breed and phenotype, we sequenced 346 DNA samples, including 121 samples (UQ, SJ, BA, Sta) from cattle with a *Bos indicus* phenotype from the NT and 225 samples from cattle of a *Bos taurus* phenotype in VIC. The D-loop regions of these samples were sequenced and aligned with reference D-loop sequences obtained from the NCBI database (*Bos taurus* mtDNA: AY676857 Angus, DQ124418 Holstein and JN817350 Chianina; *Bos indicus* mtDNA: AF492350 Zwergzebu, AY126697 Nellore and KX575711 Sahiwal) to construct a phylogenetic tree to demonstrate the relationship between the cohorts.

**Fig. 2:**
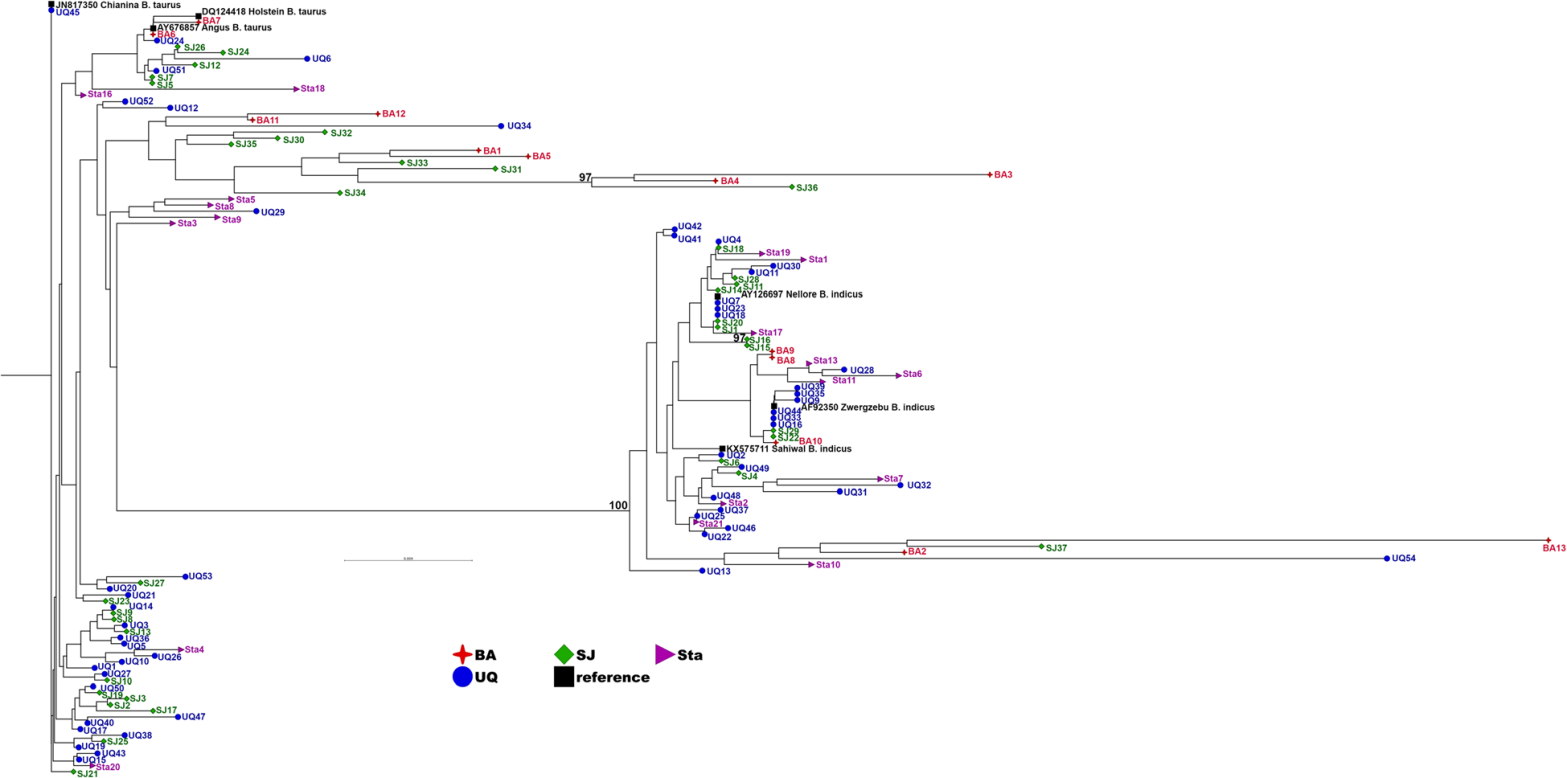
Phylogenetic tree of the D-loop regions of cattle samples from the Northern Territory, Australia. The maximum likelihood phylogenetic tree was constructed by the General Time Reversible model with 1000 bootstrap replicates using the CLC Genomics Workbench version 9.5.1. Samples were obtained from NT research and commercial breeding stations (121 samples). Each animal ID, starting with UQ, SJ, BA, Sta, refers to phenotypes listed in Table S1. UQ, SJ, BA, Sta and the reference mtDNA sequences are represented in *blue*, *green*, *red*, *purple* and *black*, respectively. Numbers above branches are bootstrap values >95%

**Fig. 3:**
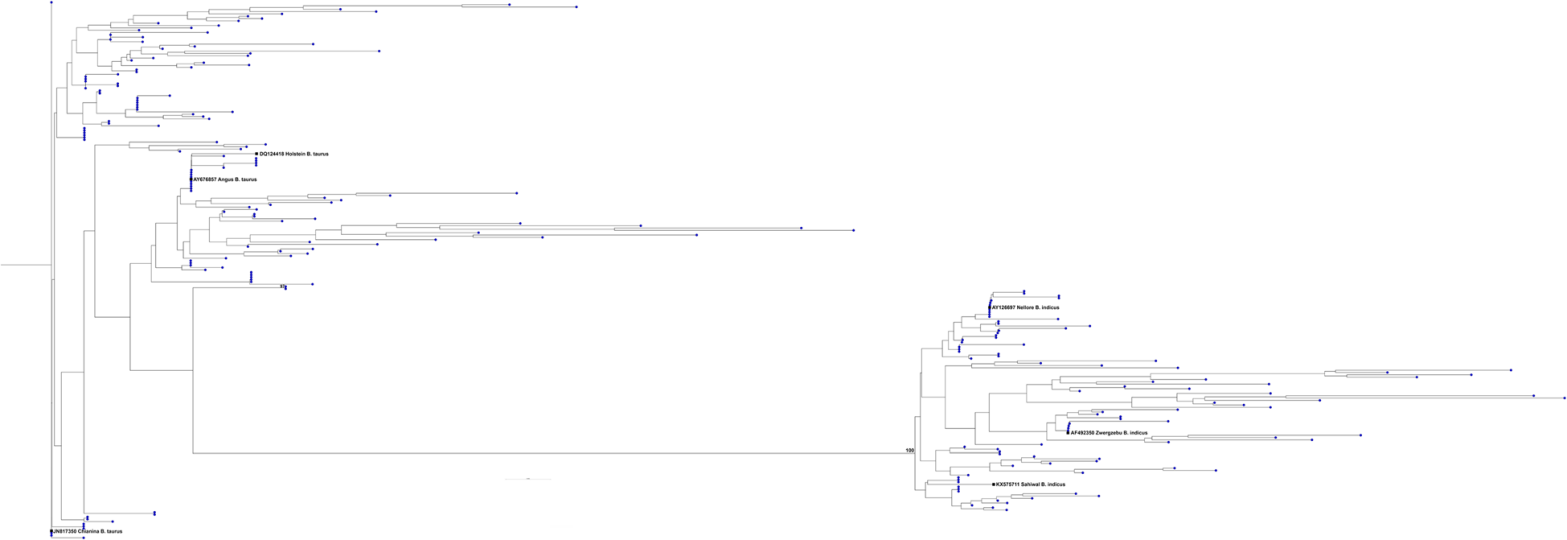
Phylogenetic tree of the D-loop regions of cattle samples from Victoria, Australia. The maximum likelihood phylogenetic tree was constructed by the general time reversible model with 1000 bootstrap replicates using the CLC Genomics Workbench version 9.5.1 to show the distribution of mtDNA haplotypes in Victoria that possessed a *Bos taurus* phenotype (225 samples). The reference mtDNA sequences are represented in *black*. Numbers above branches are bootstrap values >95%

For the NT samples with the *Bos indicus* phenotype, the phylogenetic tree, which incorporated 1000 X bootstrapping analysis, showed that the D-loop sequences formed two distinct clades with bootstrap values of 100% (Fig. 2). 54 out of 121 sequences clustered with reference *Bos indicus* mtDNA sequences (44.6%) whilst the other 67 sequences clustered with reference *Bos taurus* mtDNA sequences (55.4%). For the VIC samples with the *Bos taurus* phenotype, 133 out of 225 samples clustered with reference *Bos taurus* mtDNA sequences (59.1%). On the other hand, 92 samples clustered with reference *Bos indicus* mtDNA sequences (40.9%, Fig. 3). An additional figure file shows the phylogenetic tree of all samples from NT and VIC (see Additional file 3: Fig. S1). These results indicate that the mtDNA profiles of cattle from NT and VIC are not related to phenotype and demonstrate that they were not founded from purebred *Bos taurus* or *Bos indicus* cattle but likely from hybrids or from purebreds that underwent sperm mediated breeding programs to drive a specific phenotype.

### MtDNA copy number and embryo development of oocytes with different mtDNA profiles

**Fig. 4:**
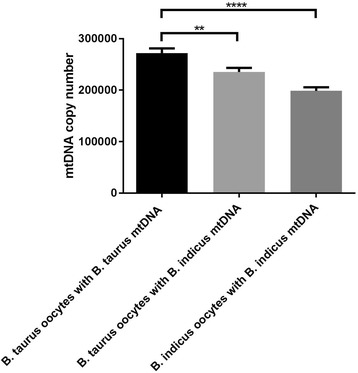
MtDNA copy number in metaphase II oocytes. *Bos taurus* oocytes harbouring *Bos taurus* mtDNA (*n* = 55) and *Bos indicus* mtDNA (*n* = 39) and *Bos indicus* oocytes harbouring *Bos indicus* mtDNA (*n* = 20) were assessed for mtDNA copy number. Mean values (± SEM) are shown. The differences were analysed for significance by one-way ANOVA with Tukey’s multiple comparisons test using GraphPad Prism version 6.01. **,**** indicate *P* < 0.01 and *P* < 0.0001, respectively

As the number of mtDNA copies in an oocyte affects fertilisation and embryo development [34], we assessed mtDNA copy number in oocytes at the MII stage. We found that mtDNA copy number in *Bos taurus* oocytes with *Bos taurus* mtDNA (271,949.66 ± 9187.40, mean ± SEM, *n* = 55) was significantly higher than that of *Bos taurus* oocytes with *Bos indicus* mtDNA (235,373.39 ± 8099.94, *P* < 0.01, *n* = 39, Fig. 4). Moreover, *Bos taurus* oocytes with *Bos taurus* mtDNA (271,949.66 ± 9187.40) had significantly higher levels of mtDNA copy number when compared with *Bos indicus* oocytes with *Bos indicus* mtDNA (198,584.09 ± 7142.37, *P* < 0.0001, *n* = 20). However, there was no significant difference in mtDNA copy number between *Bos taurus* oocytes with *Bos indicus* mtDNA and *Bos indicus* oocytes with *Bos indicus* mtDNA.

To determine the developmental potential of embryos derived from oocytes with different phenotypes and mtDNA profiles, MII oocytes were parthenogenetically activated and cultured for 7 days to generate blastocyst stage embryos. The cleavage rate for *Bos indicus* oocytes with a *Bos indicus* mtDNA profile (42.9 ± 12.1%, mean ± SEM) was significantly lower than that of *Bos taurus* oocytes with either a *Bos taurus* mtDNA profile (91.0 ± 2.2%) or a *Bos indicus* mtDNA profile (96.9 ± 3.1%, *P* < 0.05, Table 1). There were no significant differences in the developmental rates to the 8-cell and morula stages for *Bos taurus* oocytes with the *Bos taurus* and *Bos indicus* mtDNA profiles. Moreover, the blastocyst formation rate for *Bos indicus* oocytes with *Bos indicus* mtDNA (8.2 ± 4.5%) was significantly lower (*P* < 0.05) than that of *Bos taurus* oocytes with either *Bos taurus* (30.3 ± 2.0%) or *Bos indicus* mtDNA (29.2 ± 1.6%). Therefore, the developmental potential of embryos derived from *Bos indicus* oocytes with *Bos indicus* mtDNA was significantly lower than that of *Bos taurus* oocytes with either *Bos taurus* or *Bos indicus* mtDNA. However, there were no significant differences in the cleavage and developmental rates to the blastocyst stage between *Bos taurus* oocytes with *Bos taurus* and *Bos indicus* mtDNA.

**Table 1:** Developmental potential of parthenogenetically activated embryos derived from oocytes with different phenotypes and mtDNA profiles

Phenotype	mtDNA profile	No. oocyte	Cleaved (%)*	No. (%)* embryo developed to		
				8-Cell	Morula	Blastocyst
*Bos taurus*	*Bos taurus*	89	81 (91.0 ± 2.2)^a^	38 (42.7 ± 4.8)	27 (30.3 ± 42.0)	27 (30.3 ± 2.0)^a^
*Bos taurus*	*Bos indicus*	65	63 (96.9 ± 3.1)^a^	25 (38.5 ± 5.1)	23 (35.4 ± 2.8)	19 (29.2 ± 1.6)^a^
*Bos indicus*	*Bos indicus*	49	21 (42.9 ± 12.1)^b^	n/a	n/a	4 (8.2 ± 4.5)^b^

*Percentages calculated from the number of oocytes that cleaved and developed to each stage. Values are mean ± SEM

Embryos were generated from three cohorts of *Bos taurus* oocytes with a *Bos taurus* mtDNA profile and *Bos indicus* oocytes with a *Bos indicus* mtDNA profile and four cohorts of *Bos taurus* oocytes with a *Bos indicus* mtDNA profile

Different superscripts within a column indicate significant differences (*P* < 0.05, ANOVA with Duncan’s multiple range test using SAS version 9)

**Fig. 5:**
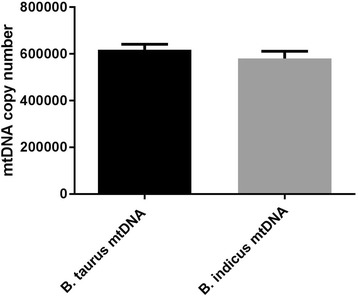
MtDNA copy number in embryos at the blastocyst stage derived from abattoir oocytes. Blastocyst stage embryos harbouring *Bos taurus* mtDNA (*n* = 18) and *Bos indicus* mtDNA (*n* = 12) were analysed for mtDNA copy number. Mean values (± SEM) are shown. No significant difference was found (unpaired t test with Welch’s correction using GraphPad Prism version 6.01)

**Fig. 6:**
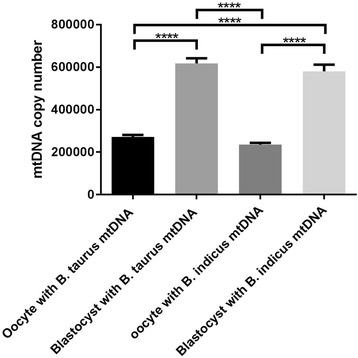
MtDNA copy number in metaphase II oocytes and blastocyst stage embryos from Victorian abattoir oocytes. Oocytes and blastocysts harbouring *Bos taurus* mtDNA (*n* = 55 and *n* = 18, respectively) and *Bos indicus* mtDNA (*n* = 39 and *n* = 12, respectively) were assessed for mtDNA copy number. Mean values (± SEM) are shown. The differences were analysed for significance by one-way ANOVA with Tukey’s multiple comparisons test using GraphPad Prism version 6.01. **** indicate *P* < 0.0001

We assessed mtDNA copy number in these embryos. MtDNA copy number in blastocysts derived from oocytes with *Bos taurus* mtDNA (617,467.36 ± 24,026.73, *n* = 18) was not significantly different when compared with that of *Bos indicus* mtDNA (580,388.70 ± 31,290.12, *n* = 12, Fig. 5). In the *Bos taurus* mtDNA group, mtDNA copy number increased from 271,949.66 ± 9187.40 in oocytes at the MII stage to 617,467.36 ± 24,026.73 in embryos at the blastocyst stage, a 2.27 fold increase (Fig. 6). Blastocysts with *Bos indicus* mtDNA had a 2.47-fold increase in mtDNA copy number (580,388.70 ± 31,290.12) when compared with that of oocytes at the MII stage (235,373.39 ± 8099.94). However, no significant difference in fold changes for mtDNA copy number between these 2 groups was found. Although oocytes from the *Bos taurus* phenotype with *Bos indicus* mtDNA had lower mtDNA copy number when compared with that of *Bos taurus* mtDNA, these oocytes had the same potential to develop to the blastocyst stage and similar levels of mtDNA in blastocysts as oocytes with *Bos taurus* mtDNA. These findings indicate that differential dependence on mtDNA between the two populations of oocytes can support oocyte competence and development to the blastocyst stage.

## Discussion

Here, we have shown that, although cattle may have one particular phenotype, such as *Bos taurus* or *Bos indicus*, it does not necessarily mean that their genetics are totally driven by this particular phenotype, or the trait that is specifically associated with a phenotype. In Australia, there is generally a ‘north-south divide’ for the various populations of cattle. For example, it is assumed that the more hardy cattle, which can tolerate parasites such as ticks and stressful tropical conditions, namely *Bos indicus* cattle, would be more suited to the tropical conditions of northern Australia. On the other hand, *Bos taurus* cattle, which include breeds such as Holstein and Angus, are more suited to the wetter and cooler climates of southern Australia. Even though southern Australia experiences high levels of heat in summer, its winters are cooler and nutrition is generally better all year round than in northern Australia. Nevertheless, our data suggest that there has been significant migration and crossbreeding of cattle from one region to the other and that cattle which appear phenotypically to look like one breed have a very different origin.

To this extent, it is evident that some of the supposed high quality Angus cattle that are associated with excellent meat quality in southern Australia have mtDNA genotypes associated with *Bos indicus*. As mtDNA is primarily maternally inherited [35], this would suggest that these Angus cattle were initially bred from females that were of *Bos indicus* origin and that the phenotype has been driven by multiple rounds of crossbreeding that have relied upon the genetic traits present within the male chromosomes. Nevertheless, a mtDNA haplotype will always persist through the female as her eggs carry only one type of mtDNA population and that is the population that she inherited from her maternal ancestors [35]. Another possibility is recombination between maternal and paternal mtDNA, a topic that has been hotly disputed in large mammals. For example, there has been one report of maternal and paternal mtDNA recombination in humans [36], although others have offered different explanations for this outcome [37]. In addition, the transmission of maternal and paternal mtDNA (non-recombination) has been reported in mice [38], sheep [39] and Drosophila [40] following interspecific crossings. Angus cows bred with indicine bulls could potentially carry maternal and paternal mtDNA, although this has not been previously reported. However, the transmission of paternal mtDNA in mice and sheep tended to be at very low levels (<5%), which suggests that our observations are indicative of maternal origins.

In the NT, we have also seen the opposite effect where some *Bos indicus* cattle carry mtDNA genomes inherited from *Bos taurus* female ancestors. This has arisen mostly because historically the herds in the NT were *Bos taurus* and, over time, many generations of crossbreeding with Brahman bulls has produced animals with a *Bos indicus* appearance. Furthermore, breeders have sought to establish breeding lines of animals that have mixed genetic profiles in order to take advantage of hybrid vigour and create animals which have as many positive phenotypic traits as possible. These include Braford (Brahman x Hereford), Brangus (Brahman x Angus), Charbray (Brahman x Charolais), Droughtmaster (Brahman x Shorthorn), and Belmont Red (African Sanga x Hereford x Shorthorn). Nevertheless, it appears that because these lines were established on a maternal profile, the maternal *Bos taurus* mtDNA population has persisted over multiple generations.

In respect of fertilisation and developmental outcomes, our results showed that *Bos indicus* oocytes had lower mtDNA copy number and their embryos had lower developmental potential, when compared with *Bos taurus* oocytes. Reports in mouse [41], pig [42–44], cattle [45, 46] and human [34, 47, 48] have shown that mtDNA copy number in oocytes plays an important role in fertilization and developmental outcomes. To this extent, fertilised oocytes have higher levels of mtDNA copy number than unfertilised oocytes, whilst oocytes with low mtDNA copy number have a lower potential to develop to the blastocyst stage. This is consistent with our results where oocytes with low mtDNA copy number had a lower blastocyst formation rate, which is likely associated low mtDNA copy number that has been observed to influence in vitro maturation rates [46]. This is further exemplified in mice where decreased postimplantation development and live offspring rates were related to oocyte mtDNA copy number [49]. As mtDNA replication in embryos does not occur until the blastocyst stage [43, 45], and is restricted to the trophectodermal cells that give rise to the placenta [43], sufficient mtDNA in the oocyte at the time of fertilisation is important to support embryo development until mtDNA replication is initiated post-gastrulation in the embryo proper [50, 51].

Moreover, our work highlights distinct differences between cattle harbouring *Bos indicus* and *Bos taurus* mtDNA genotypes indicating that the different genotypes differentially regulate developmental competence and, perhaps, development to term. Indeed, we have observed similar outcomes in commercial pigs where the oocytes from sows with different mtDNA haplotypes exhibited different levels of developmental competence and had significantly different litter sizes suggesting that each haplotype exhibited a distinct reproductive strategy [14]. Interestingly, mitochondrial supplementation of porcine oocytes with low mtDNA copy number can increase fertilization and blastocyst rates to match those of oocytes with normal levels of mtDNA, which demonstrates the importance of mtDNA in promoting developmental outcome [42, 44].

Whilst *Bos indicus* cattle often show decreased reproductive efficiency, they exhibit tolerance to heat [52] and resistance to ticks [53, 54], intestinal worms [55, 56] and buffalo fly [57] and are more efficient at utilizing low quality pastures [58] than *Bos taurus* breeds. This makes them more suitable for tropical and sup-tropical regions such as northern Australia than *Bos taurus* cattle. Generally, where environmental conditions are not stressful, *Bos taurus* outperform *Bos indicus* cattle in both growth and fertility, but where environmental conditions are stressful the performance of *Bos indicus* cattle is not reduced as much and so they perform better than *Bos taurus* cattle [59, 60]. This suggests that there is degree of evolutionary trade off where one trait is sacrificed at the expense of another [61, 62]. Nevertheless, the *Bos taurus* cattle of southern Australia harbouring *Bos indicus* mtDNA have shown adaptation by their oocytes exhibiting higher developmental competence and rates of development, which has not been observed under similar circumstances in the pig industry [14].

The influence of the mitochondrial genome over the nuclear genome is in the process of being elucidated. It is well-known that mtDNA possesses protein-encoding genes of the electron transfer chain, which generates the majority of energy for the cell. However, it has recently been demonstrated that mtDNA affects gene expression and DNA methylation as well as histone methylation patterns of the chromosomal genome [63, 64]. Moreover, defects to mtDNA can have impacts on the health of humans and animals. There are a number of diseases in humans caused by mitochondrial depletion, deletion and mutations, such as Leigh syndrome and Leber’s hereditary optic neuropathy, resulting in, for example, poor growth, developmental delays, neurological problems and muscle weakness (reviewed by [65]).

As mtDNA haplotypes have been suggested to influence milk [66] and meat [67] quality, and estimated breeding values [68] in cattle, growth and physical performance in mice [69], fertility in beef cattle [70] and pigs [42], development to term of cattle produced by somatic cell nuclear transfer [32] and expression levels of heat shock proteins in human cells [19], the combination of knowledge about mtDNA, assisted reproductive technologies and breeding strategies could lead to the generation of prime founder animals with the desired genotypes that could then be dispersed and maintained through the breeding industry. It has long been debated whether somatic cell nuclear transfer and other more sophisticated assisted reproductive technologies could be employed to generate super breeds of cattle and, specifically, new lineages of cattle (reviewed by [18, 52]). One such proposal might be to take the chromosomal traits from one breed of animal such as Holstein or Angus where there would be specific genetic markers associated with improved milk yield or meat quality, matched with mitochondrial profiles that might be associated with, for example, improved fertility or tolerance to heat or other specific traits. This can be simply achieved by taking a donor cell from one line and introducing it into the oocyte carrying a mtDNA genome of another lineage. Whether *Bos indicus* oocytes harbouring *Bos indicus* mtDNA would be appropriate hosts considering their low levels of mtDNA and developmental competence remains to be determined.

Nevertheless, it appears through chance rather than through deliberate breeding strategies, that, indeed, some of these prime animals may be present within the breeding populations in Australia. Therefore, relocation or selective breeding of superior livestock in Australia could place animals in the environments that might be optimal according to their genetic compositions. For example, those Angus cattle with the *Bos indicus* mitochondrial profiles that are associated with heat tolerance and tick resistance, could well survive in northern Australia and at the same time produce high quality meat for production purposes.

The question that remains to be answered is whether long term breeding to force a chromosomal phenotypic trait on a specific mitochondrial profile produces a meaningful outcome. What is not clear is what effect the mitochondrial genotype would have on the chromosomal phenotypic traits over multiple generations and whether the specific traits in both genomes would be continued to be propagated due to the diluting effect on the chromosomal genome from breeding.

It is well documented that the mitochondrial genome can affect gene expression patterns of the chromosomal genome as well as DNA methylation patterns of the chromosomal genome [63] along with its histone methylation patterns [64]. These are key regulators of gene expression which can determine the phenotype of the individual. In contrast, it may still be appropriate to perform somatic cell nuclear transfer where definitive phenotypic traits of the chromosome would not have been diluted out through multiple rounds of breeding. The symbiotic relationship between the chromosomal and mitochondrial genomes could be established within one round of embryo production. Moreover, live offspring production could indeed ensure that there was a greater chance for the desired trait being propagated. Only experimentation by relocating cattle from one region to another will uncover whether this is, indeed, the case. In support of breeding diluting out the effect of the mitochondrial genome, this was evident from the *Bos indicus* cattle that have adopted a *Bos taurus* phenotype where oocyte quality was similar.

## Conclusions

In all, our data highlight the importance of assessing mitochondrial profiles when planning breeding programs for cattle. It is also well known that the nuclear genome plays a major role in establishing specific traits and can be related to specific genetic markers such as the casein genes for milk production [71, 72], the myostatin gene for meat tenderness [73] and the heat shock protein genes for heat tolerance [74, 75]. However, it has been shown that mtDNA haplotypes can also affect production traits such as milk yield, meat quality, heat stress response and reproductive traits. We have shown that mtDNA haplotype affects the quality of oocytes and development of embryos. To this extent, the low developmental potential of *Bos indicus*-derived embryos correlated with low mtDNA copy number in oocytes whilst *Bos taurus* oocytes possessed significantly higher levels of mtDNA copy number and embryo development rates. Consequently, it is important that breeding programs consider not only chromosomal markers but also mtDNA markers when planning and propagating enhanced livestock herds in order to improve production efficiencies for food purposes.

## Additional files

Additional file 1: Table S1. List of breeds of cattle analysed from hair samples (DOCX 21 kb)

Additional file 2: Table S2. List of mitochondrial DNA sequences obtained from NCBI (data obtained on 31^st^ July 2016) (DOCX 14 kb)

Additional file 3: Figure S1. Phylogenetic tree of the D-loop regions of cattle samples from the Northern Territory and Victoria, Australia. The maximum likelihood phylogenetic tree was constructed by the General Time Reversible Model with 1000 bootstrap replicates using the CLC Genomics Workbench version 9.5.1. A, OVA, AE, B, MII represented cattle with a *Bos taurus* phenotype (225 samples) and are highlighted in blue. UQ, SJ, BA, Sta represented cattle with a *Bos indicus* phenotype (121 samples) and are highlighted in red. The reference mtDNA sequences are represented in black. Numbers above branches are bootstrap values >95% (PNG 844 kb)

## Abbreviations

COCs: Cumulus oocyte complexes
GTR: General Time Reversible
HKY: Hasegawa-Kishino-Yano
IVM: in vitro maturation
MII: Metaphase II
mtDNA: Mitochondrial DNA
*ND2*: NADH dehydrogenase subunit 2
NT: Northern Territory
PCR: Polymerase chain reaction
VIC: Victoria
