# Supplementary material for: Cattle phenotypes can disguise their maternal ancestry
Source: BMC Genet. 2017 Jun 26;18:59. doi: 10.1186/s12863-017-0523-5 (PMC5485690; doi:10.1186/s12863-017-0523-5)
Supplement: Supplementary file 1 — List of breeds of cattle analysed from hair samples (DOCX 21 kb) [file 12863_2017_523_MOESM1_ESM.docx]

**Table S1. List of breeds of cattle for hair samples**

| **ID** | **Breed** |
| --- | --- |
| UQ1 | Brahman |
| UQ2 | Brahman |
| UQ3 | Brahman |
| UQ4 | Brahman |
| UQ5 | Brahman |
| UQ6 | Brahman |
| UQ7 | Brahman |
| UQ9 | Brahman |
| UQ10 | Brahman |
| UQ11 | Brahman |
| UQ12 | Brahman |
| UQ13 | Brahman |
| UQ14 | Brahman |
| UQ15 | Brahman |
| UQ16 | Brahman |
| UQ17 | Brahman |
| UQ18 | Brahman |
| UQ19 | Brahman |
| UQ20 | Brahman |
| UQ21 | Brahman |
| UQ22 | Brahman |
| UQ23 | Brahman |
| UQ24 | 25% Senepol; 75% Brahman |
| UQ25 | 25% Senepol; 75% Brahman |
| UQ26 | 25% Senepol; 75% Brahman |
| UQ27 | 25% Senepol; 75% Brahman |
| UQ28 | 25% Senepol; 75% Brahman |
| UQ29 | 25% Senepol; 75% Brahman |
| UQ30 | 25% Senepol; 75% Brahman |
| UQ31 | 25% Senepol; 75% Brahman |
| UQ32 | 25% Senepol; 75% Brahman |
| UQ33 | 25% Senepol; 75% Brahman |
| UQ34 | 25% Senepol; 75% Brahman |
| UQ35 | 25% Senepol; 75% Brahman |
| UQ36 | 25% Senepol; 75% Brahman |
| UQ37 | 25% Senepol; 75% Brahman |
| UQ38 | Brahman |
| UQ39 | 25% Senepol; 75% Brahman |
| UQ40 | Brahman |
| UQ41 | Brahman |
| UQ42 | Brahman |
| UQ43 | Brahman |
| UQ44 | 25% Senepol; 75% Brahman |
| UQ45 | 25% Senepol; 75% Brahman |
| UQ46 | Brahman |
| UQ47 | Brahman |
| UQ48 | 25% Senepol; 75% Brahman |
| UQ49 | Brahman |
| UQ50 | 25% Senepol; 75% Brahman |
| UQ51 | Brahman |
| UQ52 | 25% Senepol; 75% Brahman |
| UQ53 | Brahman |
| UQ54 | 25% Senepol; 75% Brahman |
| SJ1 | Brahman |
| SJ2 | Brahman |
| SJ3 | Brahman |
| SJ4 | Brahman |
| SJ5 | Brahman |
| SJ6 | Brahman |
| SJ7 | Brahman |
| SJ8 | Brahman |
| SJ9 | Brahman |
| SJ10 | Brahman |
| SJ11 | Brahman |
| SJ12 | Brahman |
| SJ13 | Brahman |
| SJ14 | Brahman |
| SJ15 | Brahman |
| SJ16 | Brahman |
| SJ17 | Brahman |
| SJ18 | Brahman |
| SJ19 | Brahman |
| SJ20 | Brahman |
| SJ21 | Brahman |
| SJ22 | Brahman |
| SJ23 | Brahman |
| SJ24 | Brahman |
| SJ25 | Brahman |
| SJ26 | Brahman |
| SJ27 | Brahman |
| SJ28 | Brahman |
| SJ29 | Brahman |
| SJ30 | Brahman |
| SJ31 | Brahman |
| SJ32 | Brahman |
| SJ33 | Brahman |
| SJ34 | Brahman |
| SJ35 | Brahman |
| SJ36 | Brahman |
| SJ37 | Brahman |
| BA1 | Brahman |
| BA2 | Brahman |
| BA3 | Brahman |
| BA4 | Brahman |
| BA5 | Brahman |
| BA6 | Brahman |
| BA7 | Brahman |
| BA8 | Brahman |
| BA9 | Brahman |
| BA10 | Brahman |
| BA11 | Brahman |
| BA12 | Brahman |
| BA13 | Brahman |
| Sta1 | Brahman, Angus, Senepol |
| Sta2 | Brahman, Angus, Senepol |
| Sta3 | Brahman, Angus, Senepol |
| Sta4 | Brahman, Angus, Senepol |
| Sta5 | Brahman, Angus, Senepol |
| Sta6 | Brahman, Angus, Senepol |
| Sta7 | Brahman, Angus, Senepol |
| Sta8 | Brahman, Angus, Senepol |
| Sta9 | Brahman, Angus, Senepol |
| Sta10 | Brahman, Angus, Senepol |
| Sta11 | Brahman, Angus, Senepol |
| Sta13 | Brahman, Angus, Senepol |
| Sta16 | Brahman, Angus, Senepol |
| Sta17 | Brahman, Angus, Senepol |
| Sta18 | Brahman, Angus, Senepol |
| Sta19 | Brahman, Angus, Senepol |
| Sta20 | Brahman, Angus, Senepol |
| Sta21 | Brahman, Angus, Senepol |
