# Supplementary material for: Cattle phenotypes can disguise their maternal ancestry
Source: BMC Genet. 2017 Jun 26;18:59. doi: 10.1186/s12863-017-0523-5 (PMC5485690; doi:10.1186/s12863-017-0523-5)
Supplement: Supplementary file 2 — List of mitochondrial DNA sequences obtained from NCBI (data obtained on 31st July 2016) (DOCX 14 kb) [file 12863_2017_523_MOESM2_ESM.docx]

**Table S2. List of mitochondrial DNA sequences obtained from NCBI (data obtained on 31^st^ July 2016).**

| **Accession number** | **Breed** | **Species** |
| --- | --- | --- |
| KF926377 | Hanwoo | *Bos taurus* |
| EU177844 | Maremmana | *Bos taurus* |
| JN817312 | Italian Brown | *Bos taurus* |
| JN817298 | Abigar | *Bos indicus* |
| JN817344 | Italian Podolian | *Bos taurus* |
| JN817330 | Horro | *Bos indicus* |
| JN817304 | Arsi | *Bos indicus* |
| JN817307 | Creole | *Bos taurus* |
| JN817311 | Pampa Chaqueno Creole | *Bos taurus* |
| JN817300 | Alentejana | *Bos taurus* |
| JN817308 | Chihuahua Creole | *Bos taurus* |
| JN817342 | Agerolese | *Bos taurus* |
| JN817351 | Marchigiana | *Bos taurus* |
| JN817350 | Chianina | *Bos taurus* |
| EU177842 | Cinisara | *Bos taurus* |
| JN817349 | Sheko | *Bos taurus* |
| JN817305 | Boran | *Bos indicus* |
| KF163061 | Nguni | *Bos taurus* |
| KT184468 | Menofi | *Bos taurus* |
| JN817306 | Calvana | *Bos taurus* |
| EU177863 | Piedmontese | *Bos taurus* |
| KT184472 | Domiaty | *Bos taurus* |
| KP637147 | Pirenaica | *Bos taurus* |
| HQ184037 | Grey Alpine | *Bos taurus* |
| EU177867 | Cabannina | *Bos taurus* |
| HQ184035 | Romagnola | *Bos taurus* |
| EU177852 | Greek | *Bos taurus* |
| AY676856 | Limousin | *Bos taurus* |
| EU177861 | Rendena | *Bos taurus* |
| DQ124385 | Korean | *Bos taurus* |
| KT343749 | Maltese | *Bos taurus* |
| AY676857 | Angus | *Bos taurus* |
| KJ709686 | Red Mountain | *Bos taurus* |
| GQ129207 | Hungarian Grey | *Bos taurus* |
| HM045018 | Heck | *Bos taurus* |
| DQ124418 | Holstein | *Bos taurus* |
| GQ129208 | Ukrainian grey | *Bos taurus* |
| KC153977 | White Park | *Bos taurus* |
| AF492351 | Fleckvieh | *Bos taurus* |
| EU177817 | Valdostana | *Bos taurus* |
| AY676861 | Charolais | *Bos taurus* |
| EU177830 | Podolica | *Bos taurus* |
| AY676860 | Galbvieh | *Bos taurus* |
| AB074966 | Japanese black | *Bos taurus* |
| EU177834 | Betizuak | *Bos taurus* |
| GU947021 | Longhorn | *Bos taurus* |
| EU177832 | Pettiazza | *Bos taurus* |
| EU177831 | Modicana | *Bos taurus* |
| AY676855 | Simmental | *Bos taurus* |
| EU177869 | Iraqi | *Bos taurus* |
| EU177870 | Iranian | *Bos taurus* |
| AF492350 | Zwergzebu | *Bos indicus* |
| AY126697 | Nellore | *Bos indicus* |
| FJ971088 | Mongolia | *Bos taurus* |
| KT033901 | Nandan | *Bos taurus* |
| KX575711 | Sahiwal | *Bos indicus* |
